# Mining semantic networks of bioinformatics e-resources from the literature

**DOI:** 10.1186/2041-1480-2-S1-S4

**Published:** 2011-03-07

**Authors:** Hammad Afzal, James Eales, Robert Stevens, Goran Nenadic

**Affiliations:** 1 School of Computer Science, University of Manchester, Oxford Road, Manchester, M13 9PL, UK; 2 College of Telecommunication Engineering, National University of Sciences and Technology, Islamabad, Pakistan; 3 Digital Enterprise Research Institute, National University of Ireland, Galway, Ireland

## Abstract

**Background:**

There have been a number of recent efforts (e.g. BioCatalogue, BioMoby) to systematically catalogue bioinformatics tools, services and datasets. These efforts rely on manual curation, making it difficult to cope with the huge influx of various electronic resources that have been provided by the bioinformatics community. We present a text mining approach that utilises the literature to automatically extract descriptions and semantically profile bioinformatics resources to make them available for resource discovery and exploration through semantic networks that contain related resources.

**Results:**

The method identifies the mentions of resources in the literature and assigns a set of co-occurring terminological entities (descriptors) to represent them. We have processed 2,691 full-text bioinformatics articles and extracted profiles of 12,452 resources containing associated descriptors with binary and tf*idf weights. Since such representations are typically sparse (on average 13.77 features per resource), we used lexical kernel metrics to identify semantically related resources via descriptor smoothing. Resources are then clustered or linked into semantic networks, providing the users (bioinformaticians, curators and service/tool crawlers) with a possibility to explore algorithms, tools, services and datasets based on their relatedness. Manual exploration of links between a set of 18 well-known bioinformatics resources suggests that the method was able to identify and group semantically related entities.

**Conclusions:**

The results have shown that the method can reconstruct interesting functional links between resources (e.g. linking data types and algorithms), in particular when tf*idf-like weights are used for profiling. This demonstrates the potential of combining literature mining and simple lexical kernel methods to model relatedness between resource descriptors in particular when there are few features, thus potentially improving the resource description, discovery and exploration process. The resource profiles are available at http://gnode1.mib.man.ac.uk/bioinf/semnets.html

## Background

The rapid increase in the amount of bioinformatics data produced in recent years has resulted in the huge influx of bioinformatics electronic resources (e-resources), such as online-databases [1], data-analysis tools [2], Web services [3] etc. Still, many users rely on a limited set of tools that have been used and developed locally in their groups or by their collaborators, since discovering and using new resources became one of the major issues and bottlenecks in bioinformatics. Therefore, a number of community-wide efforts such as BioCatalogue [4] and BioMoby [5] have been initiated to systematically catalogue the “bioinformatics resourceome”. By collecting and annotating resources using keywords and ontological concepts, such catalogues facilitate access to both bioinformaticians and Semantic Web crawlers and agents that can orchestrate their use. However, the annotation process depends on a typically slow manual curation process that hinders the growth of such curated resources to keep pace with the very field they attempt to catalogue. For instance, the number of registered services in BioCatalogue (there were more than 1,600 of them as of March 2010) is lagging behind the total number of Web services available online: it is estimated that there are ~3500 life science Web services in Taverna alone [6]. This fact calls for the development of semi-automatic methods for resource annotation and their cataloguing in order to maximise the utility of e-resources by making them widely available to the community. 

**Figure 1:**
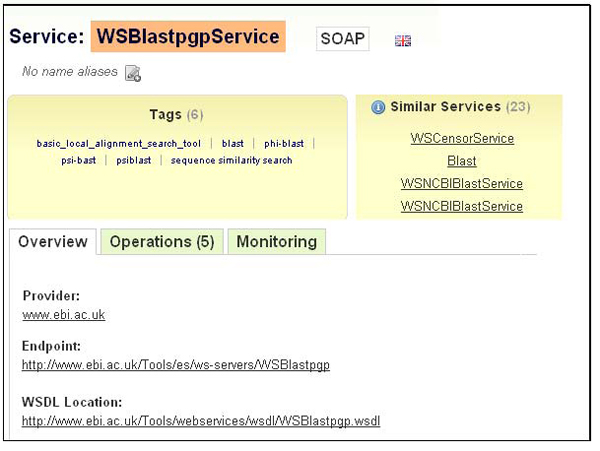
A snapshot of a Web service description taken from BioCatalogue

One of the key aims of providing bioinformatics resources with semantic descriptions is to improve resource discovery. Semantically-described resources can not only be searched, browsed and discovered by using keyword-based queries (for instance, via their names or task descriptions), but also on the basis of the semantic relatedness of their functionalities or their input/output parameters. For example, a user can search for a Web service that corresponds to a particular input, output or operation performed. If, however, the retrieved services do not fulfill the exact requirement or are not available, the user may explore similar services (for example, with more generic/specific input/output, but still with a related functionality). This process has been facilitated by *concept-based annotations* using domain ontologies that have been used to annotate the resources (as in myGrid [7] and BioCatalogue). Descriptions of services may also have “pre-computed” *similar services* (see Figure 1) so that the users can identify them without additional searches.

When manually assigned annotation tags and/or related resources are not available, we hypothesise that automated approaches could be used to improve the discovery process by generating semantic networks and clusters of similar bioinformatics resources. In this paper we propose a methodology to automatically build such networks from the literature. In our previous work, we have shown that the vast amounts of scientific literature related to bioinformatics resources can be tapped in order to automatically extract their key semantic functional features [8]. Here we do not aim to fully characterise resources (e.g. as presented in BioCatalogue), but rather to extract their descriptors that can be used to semantically link related instances. Traditionally, similar or related instances have been identified by using lexical comparisons of their names and names of their parameters (input/output) and operations. Such approaches rely on authors using similar vocabularies to name operations, parameters and messages. In order to measure semantic relatedness, in this paper we present a kernel-based similarity approach that uses lexical and semantic properties of resource mentions as extracted from the literature. Finally, we do not aim to characterise the quality or provenance of resources: the aim is to provide an exploration space for the users to discover related resources.

## Methods

The methodology is based on three concepts: mentions of bioinformatics resources, semantic resource descriptors, and similarity functions. Bioinformatics resources represent e-resources that are used by bioinformaticians while performing in-silico experiments [9]. Mentions of bioinformatics resources are identified in the literature using term identification [8]; their semantic profiles comprising semantic descriptors are also generated from the literature [8,10]; finally, the resources are inter-connected with each other on the basis of similarity between their semantic profiles that is measured using various similarity metrics.

### Identification of bioinformatics resources in text

We have focused on the four major classes of resources: *Algorithms*, *Applications*, *Data* and *Data Resources*. These have been engineered from the myGrid ontology [11]. Table 1 shows example resource instances belonging to these classes. 

**Table 1:** Examples of semantic classes and their instances

Semantic class	Example instances
Algorithm	SigCalc *algorithm*, CHAOS local *alignment*, SNP *analysis*, KEGG Genome-based *approach*, GeneMark *method*, K-fold cross validation *procedure*
Application	PreBIND Searcher *program*, Apollo2Go *Web Service*, FLIP *application*, Apollo Genome Annotation curation *tool*, GenePix *software*, Pegasys *system*
Data	GeneBank *record*, Genome Microbial CoDing *sequences*, Drug Data *report*
Data resource	PIR Protein Information *Resource*, BIND *database*, TIGR *dataset*, BioMOBY Public Code *repository*

In our previous work, we have described a set of text mining tools that can be used to identify, classify and extract mentions of e-resources in the literature [8]. The method is based on identification of key terminological heads assigned to each of the semantic classes (e.g. *alignment* and *method* are “linked” to *Algorithms*, while *sequence* and *record* point to a *Data* entity) and specific lexico-syntactic patterns (enumerations, coordination, etc.) in which such instances occur.

### Harvesting semantic descriptors

Semantic resource descriptors are the key terminological phrases used in the existing textual descriptions of bioinformatics resources to refer to concepts and specific roles (e.g. input/output parameters, etc). These have been used in the existing resource descriptions (BioCatalogue, BioMoby, EBI web services [12], etc.) to denote functionalities, dependencies, input/output constraints, etc. For example, frequent descriptors are *gene expression, phylogenetic tree, microarray experiment, hierarchical clustering, amino acid sequence, motif*, etc. We use such descriptors to profile a given resource (see below). Two sources were combined to build a dictionary of bioinformatics resource descriptors. The first source is the list of terms collected from the bioinformatics ontology used in the myGrid project. This list contains 443 terms describing concepts in *informatics* (the key concepts of data, data structures, databases and metadata); *bioinformatics* (domain-specific data sources e.g. model organism sequencing databases, and domain-specific algorithms for searching and analysing data e.g. a *sequence alignment algorithm*); *molecular biology* (higher level concepts used to describe bioinformatics data types, used as inputs and outputs in services e.g. *protein sequence*, *nucleic acid sequence*); and *tasks* (generic tasks a service operation can perform e.g. *retrieving*, *displaying*, *aligning*). The second source includes automatically extracted terms (recognised by the TerMine service [13]) and frequent noun phrases obtained from existing descriptions of bioinformatics Web resources available from BioCatalogue. 

### Semantic profiling of resources

**Figure 2:**
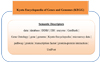
Sample of semantic resource descriptors for the Kyoto Encyclopaedia of Genes and Genomes (KEGG)

For each bioinformatics resource that is identified in the literature, we build its *semantic profile* by harvesting all descriptors that co-occur with the resource in the same sentence in a given corpus (see Figure 2 for an example). These profiles are used to establish semantic similarities between resources by comparing the descriptors (used as features) that have been assigned to them. We note that descriptors do not represent a comprehensive description of a resource, but rather an approximation extracted from the literature. Some of these descriptors may be generic (e.g. *gene*) and some very specific (e.g. *DDBJ*).

### Linking semantically related resources

We explored three methods to link semantically related resources. The first approach is based on lexical similarity between resource names (Method 1). The second approach takes into account the number of shared descriptors between resources (Method 2). However, resource representations using descriptors can be sparse (an average number of descriptors per resource is 13.77, see Table 2 in Results), in particular given a high number of potential descriptors (see Results). This suggests that it is not likely that many resources will share exactly the same descriptors. We therefore use a third approach that introduces lexical smoothing of descriptors (Method 3).

#### Method 1: lexical comparison of resource names

This method relies on lexical word-based similarity between resource names. We use the concept of *lexical profiles* to estimate similarity. The lexical profile of a term comprises all possible linear combinations of word-level substrings present in that term [14]. For example, the lexical profile of term *‘protein sequence alignment’* comprises the following terms *protein*, *sequence*, *alignment*, *protein sequence,sequence alignment*,* protein sequence alignment*. The similarity between two resources is then calculated as a similarity between lexical profiles of their names. Formally, let LP(*s*_1_) and LP(*s*_2_) be lexical profiles (represented as vectors) of names of resources *s*_1_ and *s*_2_. Then the similarity function is defined as a cosine [15,16] between vectors LP(*s*_1_) and LP(*s*_2_): 

where *< x, y>* is the inner product between vectors *x* and *y* , and |*x*| is the norm of *x*. We note that resources that share longer substrings will have a higher similarity value. 

#### Method 2: shared descriptors

Here we use the standard bag-of-descriptors approach, where each resource is represented as a bag of its descriptors and the similarity is based on exact matches between them. This method compares the resources using the inner product that measures the degree of descriptor sharing [14,15]:

where *s*_1_ and *s*_2_ are binary profile vectors that represent the semantic descriptors assigned to the resources being compared. Instead of binary weights (descriptors is/is not present in a resource’s profile), we can use a variant of term frequency – inverse document frequency (tf-*idf) weights. tf*idf is a statistical measure that is used to measure the importance of a term (or word) in a document as compared to whole collection of documents [15,16]. Here we use it to estimate how important and discriminative a given descriptor is for a given resource. We combine the relative frequency of co-occurrence of the descriptor and resource, and the inverse frequency of the descriptor with regard to all resources:

where *d* is a descriptor and *s* is a resource. Although the frequency of a common descriptor may be high, its tf*idf would be counter-balanced if it appears with a number of different resources. Each resource vector in this case comprises the tf*idf weights for all the descriptors that appear with the resource. 

Method 2 relies on resources sharing exactly the same descriptors. However, in many cases descriptors may not be exactly the same, but may be related and this should be reflected in the similarity of the associated resources. This is particularly important as the number of descriptors assigned to some resources is low, reducing the probability that other resources will have those descriptors. Therefore, we introduce an approach that takes into account the similarity between descriptors.

#### Method 3: lexical similarity of shared descriptors

In this method, we have used kernel functions to enhance the comparison process between bioinformatics resources retrieved from the literature by incorporating lexical profiles of their features. This approach is inherent to our method of employing the descriptors, as descriptors (used as features to describe resources) have been retrieved from the contextual sentences that are related to resources. Various similarity kernels can be used for comparisons (e.g. bag-of-words kernels [17,18], string kernels [19], etc.). Here we introduce a kernel function that uses lexical relatedness between descriptors to measure the similarity between the resources. The main motivation behind this approach is that resources can share related but not exactly the same descriptors. We therefore use a kernel that takes into account *descriptor smoothing* by incorporating a similarity measure between descriptors themselves in the function that calculates similarity between resources. Formally, let *S* = {*s*_1_*, …, s_k_*} be the set of e-resources whose descriptions have been collected from the literature. Let *D* = {*d*_1_*, …*,* d_m_*} be the set of all descriptors, where *m* is the total number of descriptors. In order to measure similarity between resources, we first build a descriptor similarity matrix ***A***(*m x m*), where each element a_ij_ corresponds to the similarity between descriptors *d*_i_ and *d*_j_. This similarity is calculated as the cosine between the lexical profiles of the descriptors. More precisely, 

Then, the similarity between two resources *s_1_* and *s_2_* is calculated as:

where 

Note that vectors *s_1_* and *s_2_* are normalised and can contain either binary or tf*idf feature weights. 

**Figure 3:**
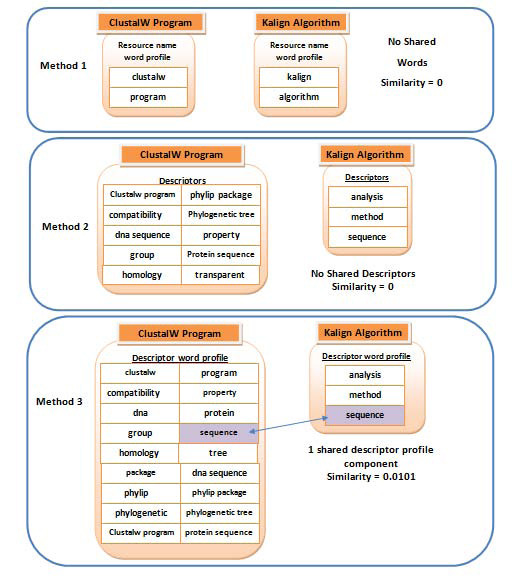
**Example of measuring similarities** between two bioinformatics resources (*Kalign Algorithm* and *ClustalW program*) using the three methods of similarities: lexical similarity between the resource names, shared descriptors between the resources, and shared descriptors between resources after their lexical smoothing.

Figure 3 shows an example of two resources and their similarities calculated using the three methods. It demonstrates the utility of using semantic descriptors of resources, and employing the kernel-based similarity functions to measure the semantic relatedness between bioinformatics resources.

## Results

Here we demonstrate the development of networks of related resources by using each of the three methods introduced above. The networks are visualised as weighted, undirected graphs where nodes are resources and edges represent relatedness between them. This *relatedness* is estimated using the three similarity functions (as explained in Methods), where the weight of an edge represents the strength of the relationship between the two connected nodes. We also investigate different methods of exploring and visualising our similarity matrices. Specifically, we use hierarchical clustering dendrograms, heatmap visualisations and semantic networks.

### Data

Table 2 gives the number of bioinformatics resources that were identified in a corpus of 2,691 full-text articles published by the journal *BMC Bioinformatics*. The details of the extraction process are given in [8].

We extracted a total of 12,452 e-resources and 1,518 descriptors. Table 3 presents the most frequent single word, two- and three-word descriptors. Each of the e-resources has been assigned a set of associated descriptors (13.77 descriptors on average; see Table 2 for details for the specific classes). 

**Table 2:** The statistics of bioinformatics e-resources found in the *BMC Bioinformatics* corpus

Semantic Class	Total number of instances	Average number of descriptors
Algorithm	5,722	11.47
Application	2,076	10.38
Data	2,662	18.77
Data Resource	1,992	12.94

**Table 3:** The most frequent single-word, two-word and three-word descriptors, along with their total frequency in the corpus

Single word descriptors	Two-word descriptors	Three-word descriptors
*gene*: 13,585	*gene expression*: 1,147	*protein-protein interaction*: 308
*method*: 8,203	*secondary structure*: 887	*multiple sequence alignment*: 295
*protein*: 6,417	*protein sequence*: 780	*gene expression data*: 262
*sequence*: 5,991	*protein structure*: 574	*amino acid sequence*: 257
*analysis*: 4,287	*microarray experiment*: 488	*Smith-Waterman algorithm*: 48

### Exploration of Semantic Networks of Bioinformatics Resources

Here we assess the utility of resource descriptors for semantic profiling and linking of bioinformatics resources. We do this by exploring our hypothesis that bioinformatics resources can be semantically linked via resource descriptions. We do not aim to evaluate individual semantic profiles and the quality of extracted descriptors, but rather the usefulness of links based on them. For this we have manually identified an evaluation sample of 18 resources that are commonly used in bioinformatics (see Table 4) and that we have extensive experience with. The sample contains resources from all four resource classes, and each of these has occurred in more than 120 sentences in our corpus. The aim of the evaluation was to establish whether the literature-extracted links correspond to semantic relatedness between the resources, i.e. if groupings of the 18 resources reflect their roles and functions. The limited size of the evaluation set allowed us to comprehensively analyse and examine all links and learn lessons on a set of familiar resources through thorough link and cluster analyses that were performed by a domain expert (JE). 

**Table 4:** A sample of resources used for exploration

Resource name	Resource class	Number of Sentences	Number of descriptors
*Gene ontology (GO)*	Data resource	6757	289
*Support vector machine (SVM)*	Algorithm	2456	134
*Protein data bank (PDB)*	Data resource	904	102
*Hidden Markov model (HMM)*	Algorithm	602	94
*Principal components analysis (PCA)*	Algorithm	599	18
*Position-specific scoring matrix (PSSM)*	Algorithm	457	24
*Self organising map (SOM)*	Algorithm	305	137
*Medical subject headings (MeSH)*	Data resource	261	138
*Neural network*	Algorithm	256	158
*Markov chain Monte Carlo (MCMC)*	Algorithm	252	132
*Expression profile*	Data	252	136
*Basic local alignment search tool (BLAST)*	Application	238	160
*Phylogenetic tree*	Data	233	175
*Structural classification of proteins (SCOP)*	Data resource	216	114
*Kyoto encyclopaedia of genes and genomes (KEGG)*	Data resource	187	143
*Clusters of orthologous groups (COG)*	Data resource	163	94
*ChIp-chip data*	Data	126	66
*Pairwise alignment*	Data	123	80

The data has been generated using the three methods for deriving semantic relatedness between resources as described above. However, the results and similarities presented here are restricted to the selected 18 resources.

**Method 1: lexical comparison of resource names**. As expected, this method did not yield useful results as very little similarity was found between resource names, in particular in smaller sets of resources. Non-zero similarity was only obtained between *Protein data bank* and *ChIp-chip data* (similarity of 0.28) and *Basic local alignment search tool* and *Pairwise alignment* (0.18). 

**Figure 4:**
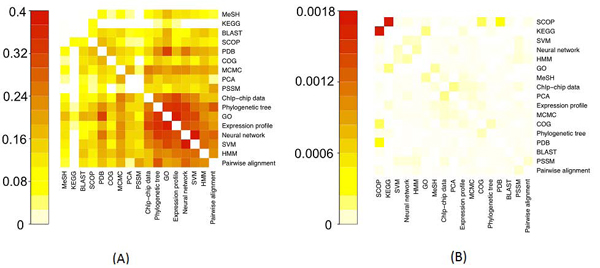
**Heatmap representations** of the matrix of shared descriptor similarity scores between resources (method 2). (A) The scores based on binary weights. (B) The scores based on tf*idf. Heatmaps generated by R function ‘heatmap’ [20]. Note that the heatmap diagonals (self-similarity) are intentionally left white to make them easier to interpret, and that the heatmaps are different scales.

**Method 2: shared descriptors**. We derived mutual similarity scores for the 18 resources based on shared semantic descriptors. Two experiments were performed: one with binary-valued features and one with tf*idf weights. In both cases, this method identified significant relatedness between many resources (see Figure 4 for heat-maps). Clearly, the addition of descriptors improved our ability to derive a measure of semantic similarity between related resources whose names are lexically disparate. However, while the binary-weighted scores brought many similarities between a number of resources (making it difficult to define any clear semantic relationships from these data), the tf*idf scores were significantly more discriminative (see Figure 4B), clearly highlighting related resources. 

**Figure 5:**
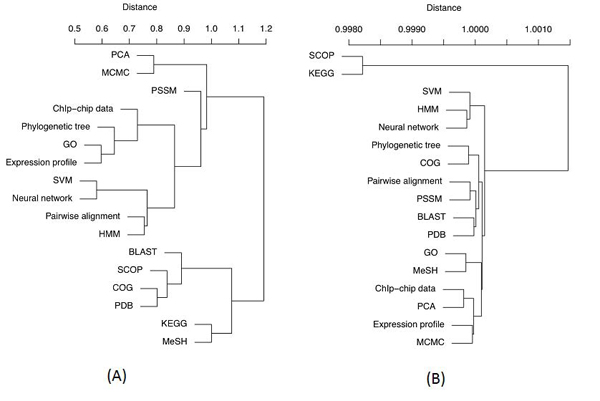
**Hierarchical clustering of e-resources** using the shared descriptors similarity matrix (method 2). (A) The scores based on binary weights. (B) The scores based on tf*idf. Distances were calculated as (1 – Sim_2_). Ward’s minimum variance clustering method [21] was used to cluster the data. The tree was generated using R function ‘hclust’ [20].

To further highlight the subtle differences and similarities between the resources in the sample, we applied a hierarchical clustering algorithm [21] to the two matrices of scores. The tree in Figure 5A highlights some interesting clusters of the examined resources when only binary indication of descriptors’ presence was used. Binary weights provided a spread of similarity scores, which better suited hierarchical clustering. In the resulting dendrogram, many resources have been grouped together based on their class (e.g. *PCA* and *MCMC* are algorithms; *COG* and *PDB* are data resources as well as *KEGG* and *MeSH*). However, the cluster of *pairwise alignment* and *HMM* highlights the semantic theme of sequence analysis. It is interesting that *BLAST* was not linked to these, while it makes a protein-related cluster with *COG*, *SCOP* and *PDB*. Furthermore, *KEGG*, *BLAST*, *COG*, *SCOP*, *PDB* and *MeSH* form their own group, which does not highlight any obvious semantic relationships; a likely reason is that these resources represent very common and fundamental resources in bioinformatics, so share quite a large group of generic descriptors.

While providing a flatter structure, Figure 5B highlights more reliable associations, typically between data resources and algorithms. For example, *BLAST* and *PDB* are closely related, as are *pairwise alignment* and *PSSM*, highlighting the semantic theme of sequence analysis. Together, *pairwisealignment*, *PSSM*, *BLAST* and *PDB* make a group that share a theme of being related to sequence analysis. *Phylogenetic tree* and *COG* form their cluster (COG is an attempt to phylogenetically group proteins [22]). It is also interesting that *HMM*, *SVM* and *neural networks* are all grouped together, representing a machine learning theme (classifiers), while *GO* and *MeSH* make their own cluster as the only semantic hierarchical resources in the set, which are typically used for annotations. 

**Figure 6:**
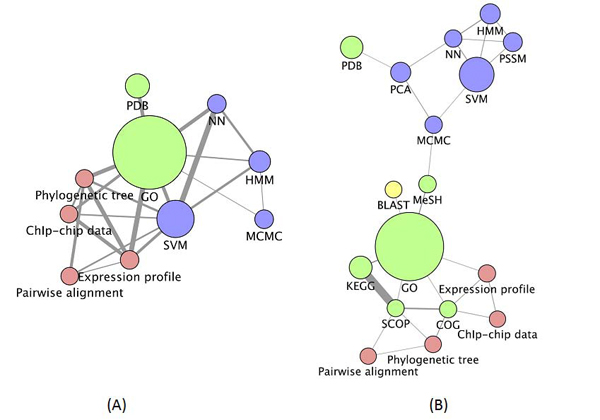
**Semantic network of bioinformatics** resources (using method 2 and values shown in Figure 4). Node size represents frequency in the corpus; edge thickness represents how similar the two connected nodes are. Node colour is determined by the semantic class of the node: red for *Data*, green for *Data resource*, blue for *Algorithm* and yellow for *Application*. (A) The scores based on binary weights. (B) The scores based on tf*idf. The image was generated using Cytoscape [23], the network was laid out using the Cytoscape layout algorithm ‘Edge-Weighted Spring Embedded’, using the edge weight data in the network.

Even though similarity data alone can identify important semantic links, we further explored the importance of the number and strength of links between resources. In Figure 6 we present the similarity data as edges in a network connecting each node (representing individual resources) with those that have some similarity to it. Each edge is weighted by the similarity between the resources it connects, so that edges that appear thick represent strong relationships and weak relationships are represented by thin edges. We have removed all edges that have a weight below the median edge weight for the network, or below the median weight for a given node. Nodes that are left with no edges are not presented in the resulting networks. Our intention with this was to remove edges that exist due to chance alone and to better highlight the strongest relationships in the network. 

The strongest links in Figure 6A are between *HMM*, *SVM* and *neural network*, identifying the machine learning theme. There is also a strong link between *Gene Ontology* and *PDB*, reflecting the fact that PDB identifiers are mapped to the GO terms. *Gene Ontology* and *SVM* are also strongly linked, most probably because *SVM* methods have been widely used for protein annotations using GO (see, for example, [24]). Figure 6B (based on tf*idf) brings all algorithm instances into a sub-network. There is also a sub-network related to sequence analysis.

**Figure 7:**
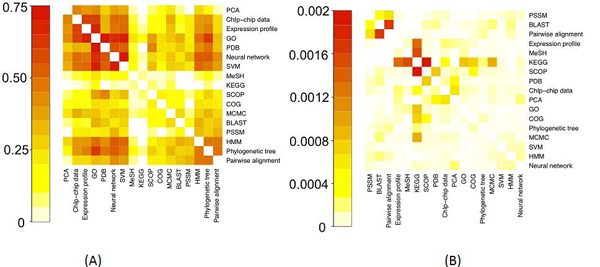
**Heatmap representation** of the matrix of lexically smoothed descriptor similarity scores between resources (method 3). (A) The scores based on binary weights. (B) The scores based on tf*idf. Heatmaps generated by R function ‘heatmap’ [20]. Note that the heatmaps are represented using different scales.

**Figure 8:**
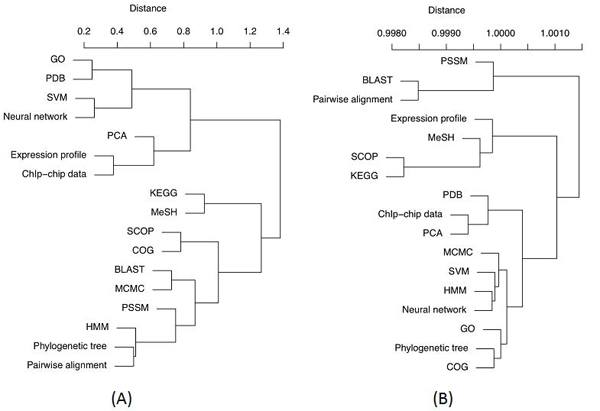
**Hierarchical clustering** of e-resources using the lexically smoothed descriptor similarity matrix (method 3). (A) The scores based on binary weights. (B) The scores based on tf*idf. Distances were calculated as (1 – Sim_3_). Ward’s minimum variance clustering method [21] was used to cluster the data. The tree generated using R function ‘hclust’ [20].

**Figure 9:**
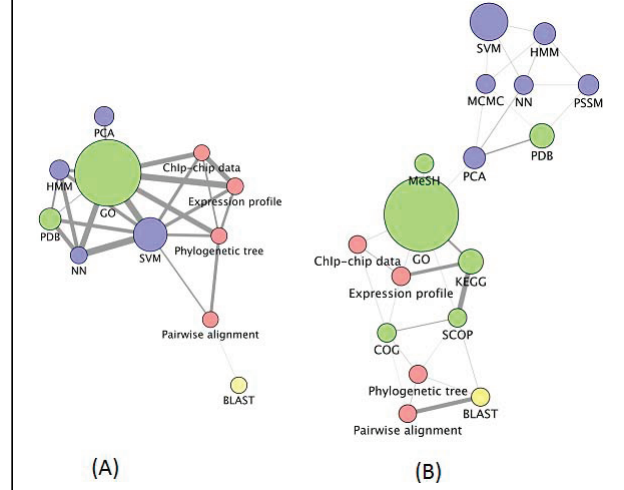
**Semantic network of bioinformatics** resources (using method 3 and values shown in Figure 7). Node size represents frequency in the corpus; edge thickness represents how similar the two connected nodes are. Node colour is determined by the semantic class of the node: red for *Data*, green for *Data resource*, blue for *Algorithm* and yellow for *Application*. (A) The scores based on binary weights. (B) The scores based on tf*idf. The image was generated using *Cytoscape* (see Figure 6 for further details).

**Method 3: lexical similarity of shared descriptors**. The results of calculations for linking the resources considering the lexical similarities between their descriptors are summarised in figures 7, 8 and 9. 

Figure 7 has many similarities with Figure 4. As before, the tf*idf scores were more selective in linking resources than binary features. However, as expected, descriptor smoothing has introduced more similarities than Method 2 in tf*idf-based similarities (compare figures 4B and 7B). 

Figure 8A shows an informative cluster made of *PCA*, *Expression profile* and *ChIp-chip* (*PCA* is a commonly used method to analyse both protein expression data (expression profiles) and *ChIp-chip* data). Data resources *phylogenetic tree* and *pairwise alignment* have been clustered together, both of which are common data forms in sequence analysis. *GO-PDB* and *SVM-neural network* also made their own groupings. Links in Figure 8B bring together another cluster related to sequence analysis group (*BLAST*, *pairwise alignment* and *PSSM*). There is again a clear sub-tree with machine learning classifiers.

The networks given in Figure 9 present the strongest grouping of resources based on their class. *Data* nodes (represented in red) and *Algorithm* nodes (blue) are strongly linked to each other. The strongest edge weights again occur between resources that appear most frequently in the corpus, suggesting that frequency normalisation may be needed to reduce this impact. *Gene Ontology*, in particular, is linked to all other resources, and that is primarily a product of its ubiquity in the literature and therefore the tendency for many descriptors and resources to be linked to it. A very strong link between *pairwise alignment* and *BLAST* was only highlighted using the tf*idf weights (Figure 9B).

## Discussion

In order to establish similarity between resources, their literature-based profiles are compared using three levels of representations: the lexical similarity between resource names (method 1); the similarity calculated on the basis of shared semantic descriptors (method 2), and the same similarity smoothed by considering lexically similar descriptors (method 3). As expected, the first method failed to capture any implicit links between resources as it relied solely on the surface level clues originating from the names of resources. Of course, in a larger set it is likely that some resources will be lexically linked, but many non-lexical links would be missed. The second approach performed better in that sense, and was able to identify interesting clustering patterns between the resources that did not have any lexical resemblance. At the third level, in contrast to considering the exact match between resource descriptors, we devised a descriptor-based kernel matrix that incorporated the approximate lexical similarities between the descriptors (using their lexical profiles). The approximate similarities helped in linking the resources that shared the descriptors that were not exactly the same, but were related (see Figure 3). By further analysing the associated semantic profiles, we can see that significant relatedness between resources typically originates from sharing a number of generic descriptors, in particular single-word ones (see Table 3). Many of these have a generic nature (e.g. *method*, *analysis*, *gene*, etc.) and are not discriminatory enough for establishing semantic relatedness at non-generic levels. This problem was addressed through using tf*idf-based scoring weights assigned to descriptors (considering the frequency of descriptors appearing in profiles of different resources), which resulted in more informative and semantically-relevant groupings of resources. Previous results in annotation of texts (and entities modelled by text features) with various categories (including ontological structures) have shown that tf*idf was typically a measure of choice and outperformed other measures by discriminating features that have been over-represented [15,16]; for example, it was widely used to annotate protein function with concepts from the Gene Ontology [25-28]. 

Semantic networks generated from the literature can be useful for both bioinformaticians that are exploring resources corresponding to their needs, and resource curators, who could accelerate their work by discovering and annotating sets of related resources. For example, an interesting pattern emerged whilst experimenting with Method 3, whereby resources would cluster together based on their class (i.e. the resources that belonged to the same class (such as *Algorithm*, *Data Resource*, etc.) tended to appear closer in the network). On the other hand, Method 2 revealed some interesting functional links (linking data types and algorithms). It remains an open question as to which of these clustering patterns is most useful for semantic resource discovery and/ or curation. Selecting and varying the threshold for the edge weight in our network representations can also discard weaker links – we have experimented with discarding all edges that are below the global or local median value. We plan to further explore improving the querying of resource profiles by organising them in an RDF-store that would facilitate retrieval of related resources, and then to test these in curation and service discovery tasks. Of course, this would include developing resource identification, normalisation and disambiguation techniques, as some resources may appear under different names/acronyms. We also note that better coverage would be possible if anaphoric expressions in sentences are resolved.

### Related work

The domain of life sciences has witnessed many efforts in the direction of utilizing Semantic Web technologies, where particular focus has been on data annotation (e.g. a number of protein function databases), using both manual and automated approaches. These efforts have recently been extended to semantic description of resources (e.g. services and tools) that are used to analyse, explore and visualise such data. These approaches include assigning meta-data about functionalities, inputs and outputs. The majority of automatic approaches to service annotation rely on the data available in *Web Service Description Language* (WSDL) files associated with Web services. Such files describe programmatic interfaces to services, including data types, input and output message formats and the operations provided. For example, Lerman and colleagues [29] presented work on automatic labelling of input and output of Web services using meta-data based classification relying on terms extracted from the associated WSDL files. The underlying heuristic behind the meta-data based classification is that similar data types tend to be named by similar names and/or belong to operations or messages that are similarly named. Similarly, Hess and Kushmerick [30] used machine learning to classify Web services using information given in WSDL files of the services that include port types, operations and parameters along with any documentation available about the Web service. Information in a WSDL file is treated as normal text, and the problem of Web service and its metadata classification is addressed as a text classification problem. Liu and Wong [31] used clustering to identify homogenous service “communities”, where features were also extracted from associated WSDL files. They also use simple text processing and statistics to identify content-describing terms, and argue that clustering services in functional groups can facilitate more effective service discovery. 

Carman and Knoblock, on the other hand, reported on invoking new/unknown services and comparing the data they produce with that of known services, and then use the meta-data associated with the known services to add annotations to the unknown resources [32]. Belhajjame and colleagues [33] used known annotations of parameters belonging to components in a workflow to infer the unknown annotations of other parameters (in other components). Here, semantic information of operation parameters is inferred based on their connections to other (annotated) components within existing tried-and-tested workflows. Apart from deriving new annotations, this method can inspect the parameter compatibility in workflows and can also highlight conflicting parameter annotations. Similarly, Dong et al. [34] used clustering-based approach in which parameters of service operations were used to find similar services. 

To the best of our knowledge, the work reported here is the first attempt to extract semantic networks of bioinformatics resources from the literature. Since the number of features in our approach for many instances was small, we have used kernels to “expand” the feature space by taking into account feature similarities. This is similar to latent semantic kernels (LSK, [35]) and semantic smoothing [36]. These methods also try to bypass the problem of exact matching by deriving “conceptual” indices using either statistical analysis of word co-occurrence across documents (LSK) or information from a static, external semantic network (semantic smoothing). Our method, on the other hand, relies on dynamic lexical similarities between features. We note that matrix A in formula (5) can be generated in different ways (as long as it is positive semi-definite i.e. represents a kernel [35]). Future work, therefore, will explore combining various kernels to build this similarity matrix, including combination of LSK and similarities based on the myGrid ontology.

## Conclusions

In this paper we proposed and explored a literature-based methodology for building clusters and semantic networks of functionally related e-resources in bioinformatics. The main motivation is to facilitate the resource discovery approaches that would improve the availability and utility of these resources to the community. The methodology revolves around semantic descriptors that are frequently used by bioinformatics resource providers to semantically describe the resources. The semantic descriptors have been automatically compiled and each e-resource has been assigned a set of descriptors co-occurring with the given e-resource in a full-text article corpus. As a proof-of-concept, the approach was evaluated on a subset of manually selected resources that the authors were familiar with. The results suggest that the method was able to group and link semantically related entities. Semantic networks that were based on tf*idf-weights in particular were more informative in “recovering” semantic relatedness between the resources. We envisage that such semantic networks will be useful to both bioinformaticians who are exploring and discovering new resources, and resource curators (e.g. in the BioCatalogue project). Furthermore, by providing an RDF-store of extracted profiles, tools and services can be integrated and queried together with the rest of the “bioinformatics resourceome”, in particular as part of service/data search engines and crawlers.

One of the major issues with the literature-based approach to resource profiling is that many resources do not appear frequently and are represented by small descriptor sets. Therefore, we explored expanding the feature space by using the “kernel trick” [35], where (lexical) similarity between features is taken into account when calculating similarity between instances. The results demonstrate the potential of even simple kernel methods (using lexical profiles) to model relatedness between resource descriptors. We anticipate that further work will be required to explore the most relevant weights for semantic descriptors to counter-balance the impact of frequent (and less informative) features. Other kernels (such as contextual and distributional similarities, ontology-based similarities, string kernels etc.) need to be explored and could provide better resolution of the complex interrelationships between features (descriptors) and consequently between bioinformatics resources. Finally, further studies would be needed to establish which methods would be most suited in supporting resource curation and discovery tasks.

## Competing interests

The authors declare no competing interests.

## Authors' contributions

HA implemented the literature mining modules; HA and JE developed and applied kernel metrics; JE performed data analysis and evaluation; RDS and GN conceived and supervised the study. The manuscript was initially drafted by HA and JE. All authors read and approved the manuscript.
